# Weak cation exchange magnetic beads coupled with matrix-assisted laser desorption ionization-time of flight-mass spectrometry in screening serum protein markers in osteopenia

**DOI:** 10.1186/s40064-016-2276-4

**Published:** 2016-05-21

**Authors:** Wei-Tao He, Bo-Cheng Liang, Zhen-Yu Shi, Xu-Yun Li, Chun-Wen Li, Xiao-Lin Shi

**Affiliations:** The Second Clinical Medical College, Zhejiang Chinese Medical University, Hangzhou, 310005 China; Department of Diagnostics of Traditional Chinese Medicine, College of Basic Medical Science, Zhejiang Chinese Medical University, Hangzhou, 310005 China; Department of Osteology, The Second Affiliated Hospital of Zhejiang Chinese Medical University, Hangzhou, China

**Keywords:** Osteopenia, Weak cation exchange magnetic beads, Proteomics, Biomarkers

## Abstract

The present study aimed at investigating the weak cation magnetic separation technology and matrix-assisted laser desorption ionization-time of flight-mass spectrometry (MALDI-TOF-MS) in screening serum protein markers of osteopenia from ten postmenopausal women and ten postmenopausal women without osteopenia as control group, to find a new method for screening biomarkers and establishing a diagnostic model for primary type I osteoporosis. Serum samples were collected from postmenopausal women with osteopenia and postmenopausal women with normal bone mass. Proteins were extracted from serum samples by weak cation exchange magnetic beads technology, and mass spectra acquisition was done by MALDI-TOF-MS. The visualization and comparison of data sets, statistical peak evaluation, model recognition, and discovery of biomarker candidates were handled by the proteinchip data analysis system software(ZJU-PDAS). The diagnostic models were established using genetic arithmetic based support vector machine (SVM). The SVM result with the highest Youden Index was selected as the model. Combinatorial Peaks having the highest accuracy in distinguishing different samples were selected as potential biomarker. From the two group serum samples, a total of 133 differential features were selected. Ten features with significant intensity differences were screened. In the pair-wise comparisons, processing of MALDI-TOF spectra resulted in the identification of ten differential features between postmenopausal women with osteopenia and postmenopausal women with normal bone mass. The difference of features by Youden index showed that the highest features had a mass to charge ratio of 1699 and 3038 Da. A diagnosis model was established with these two peaks as the candidate marker, and the specificity of the model is 100 %, the sensitivity was 90 % by leave-one-out cross validation test. The two groups of specimens in SVM results on the scatter plot could be clearly distinguished. The peak with m/z 3038 in the SVM model was suggested as Secretin by TagIdent tool. To provide further validation, the secretin levels in serum were analyzed using enzyme-linked immunosorbent assays that is a competitive inhibition enzyme immunoassay technique for the in vitro quantitative measurement of secretin in human serum.

**Electronic supplementary material:**

The online version of this article (doi:10.1186/s40064-016-2276-4) contains supplementary material, which is available to authorized users.

## Background

Osteoporosis is a skeletal disorder characterized by low bone mass and bone microstructure degeneration. With the advent of the aging society, the incidence rate of osteoporosis is rising quickly, osteoporosis has become a serious threat to the health of the elderly (Consensus NIH 2001; Czerwiński et al. 2006).

Before people develop osteoporosis, they have a condition called osteopenia. Osteopenia was firstly defined by the World Health Organization (WHO) in June 1992(Knapp et al. 2004). Osteopenia usually doesn’t cause any symptoms. Losing bone mass is not painful. Broken bones or fractures can occur, but these problems tend to happen after osteoporosis has developed. Osteopenia is not a disease, but if you meet the criteria for osteopenia, you are at higher risk for developing osteoporosis. Early diagnosis and treatment can prevent osteopenia from becoming osteoporosis.

The bone mineral density (BMD) measurement is useful in clinical application on the diagnosis of osteoporosis and the prediction of fracture in elderly women, but it is a lagging-not an early-indicator. Dual-energy X-ray absorptiometry (DXA) is currently recognized as gold standard of osteopenia and osteoporosis diagnosis (Kanis et al. 2013). Although DXA measurement is the gold standard, but early symptom of osteopenia or osteoporosis is not easy to detect, so that many patients with osteopenia or osteoporosis are often found after the first fracture. BMD does not reflect the change of bone matrix, bone turnover and bone strength. BMD could not accurately predict the risk of bone fractures (Oei et al. 2013).

With the completion of human genome project, the post genomic era is coming. We can rapidly screen the specific biomarkers of the disease by proteomics technologies (Ray et al. 2011), clarify the pathogenesis of the disease, and explore new methods of preventing and treatment.

Magnetic beads with large separation capacity has been widely used in cancer research using serum samples and beads based technology enrichment are high-throughput, simple and quick to operate (Wu et al. 2012; Fan et al. 2012).

Matrix-assisted laser desorption/ionization-time of light-mass spectrometry (MALDI-TOF-MS) is a recently emerged proteomics method, with the potential to detect various clinical samples, such as serum, urine, pleural effusion, ascites and a number of secretions (Zhang et al. 2012; Zheng et al. 2011). Analysis of the serum samples proteome may detect changes that reflect bone issues with pathological processes in the bone turnover. Furthermore, serum samples are simply and non-invasively obtained. Thus, proteomic analysis of serum samples may be a useful tool in the prediction, early diagnosis, treatment monitoring, and prognostic assessment of osteopenia patients. Weak cation exchange magnetic beads (MB-WCX) combined with MALDI-TOF-MS is one such approach that offers a unique tool for profiling of proteins, but this approach has not been used in the osteopenia area.

In this study, we introduced a developed optimal method of mass spectrometry-based technology, weak cation magnetic separation technology combined with MALDI-TOF-MS, searching for efficient serum protein biomarkers, attempting to predict Molecular mechanisms of osteopenia, thus reducing the uncertainties and potential risks in the primary type I osteoporosis patients. In this study, we developed a strategy for screening serum proteins <20 kDa to analyze serum profiles and find potential biomarkers for the osteopenia.

## Results

### Statistical analysis of baseline data

There are no statistically significant difference (P > 0.05) between comparisons of subjects of age, weight, height, and duration of menopause and it’s comparable. The results were shown in Table 1.

**Table 1:** Comparisons of clinical features of patients

Groups	Age (years)	Weight (kg)	Height (cm)	Duration of menopause (years)
Osteopenia (n = 10)	56.32 ± 3.61	53.16 ± 5.36	160.50 ± 10.53	5.26 ± 2.61
Normal bone mass (n = 10)	55.00 ± 3.48	52.24 ± 3.97	161.48 ± 11.00	4.76 ± 1.57
t	0.83	0.44	0.20	0.52
P values	>0.05	>0.05	>0.05	>0.05

Data shown are mean ± standard deviation

### Detection of differential features

**Fig. 1:**
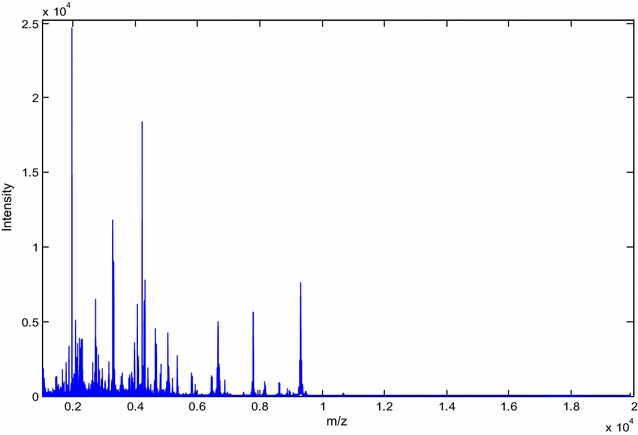
Detection of differential features. The spectrum shown in Fig. 1 is a composite spectrum from all spectra obtained from all individual patients

By application of WCX combined with MALDI-TOF MS, we analyzed the proteomic profiles between two groups. The serum samples contain a high diversity of proteins. A total of 133 peaks in the molecular weight range of 1000–20,000 Da were identified between the two groups in this study. The results are shown in Fig. 1.

### Discriminative ability of representative differential features

**Fig. 2:**
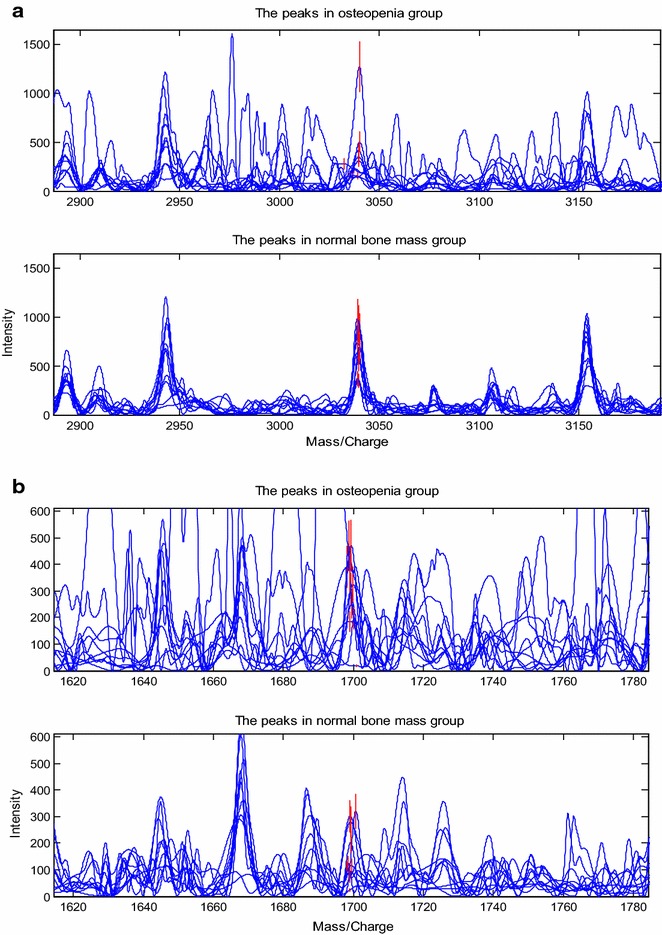
Discriminative ability of representative differential features. The top 2 discriminating peaks of 1699 and 3038 Da could distinguish serum samples between osteopenia and postmenopausal women with normal bone mass effectively. **a** Displayed the peaks of 3038 Da. **b** Displayed the peaks of 1699 Da

Genetic algorithm (GA) and support vector machine (SVM) embedded in ZJU-PDAS software (Qiu et al. 2009) were used to establish cross-validated classification model for distinguishing osteopenia group from normal bone mass group. Although there were 10 peaks showing different peaks between two groups (P < 0.05), only two (1699 and 3038 Da) of them showed statistically significant differences in further analysis by Youden Index (Fluss et al. 2005; Bantis et al. 2014). The peak mass, P values, average peak intensity, and standard deviation (SD) of the four peaks were shown in Table 2. The peaks analyzed to generate a classification model were based on the two peaks with m/z of 1699 and 3038 Da in Fig. 2. In the optimal SVM model, the two peaks with molecular weight of 1699 Da were upregulated in patients with osteopenia and 3038 Da were downregulated.

**Table 2:** Statistics of significantly different expressed peak intensities to distinguish patients from controls

m/z	Osteopenia group	Normal bone mass group	P vaules	Expression change
1699	299.04 ± 140.72	164.32 ± 105.04	0.037	↑
3038	383.78 ± 332.46	649.97 ± 236.04	0.014	↓

Data shown are mean ± standard deviation

### Development of diagnostic models and evaluation of their diagnostic accuracy

**Fig. 3:**
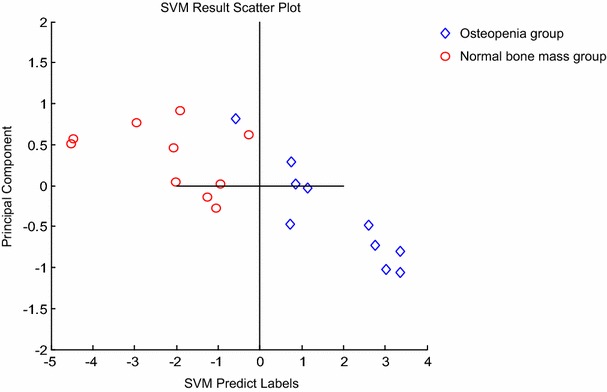
Development of diagnostic models and evaluation of their diagnostic accuracy. SVM result scatter plot, each point represents a sample, *x-axis* a main component; *y-axis* the predicted value. Mass/change means m/z

The top peaks of 1699 and 3038 Da could distinguish serum samples between osteopenia group from normal bone mass group effectively. The results are shown in Table 3. The serum proteomic model, based on a group of 2 peaks, accurately recognized osteopenia group from normal bone mass group. A diagnosis model was established with these two peaks as the candidate marker. Two groups of specimens could be clearly distinguished in the SVM result scatter plot. The result is shown in Fig. 3.

**Table 3:** Diagnostic accuracy of different classification models in the training set

Groups	Predicted osteopenia	Predicted normal bone mass	Sum	Accuracy (%)	Error (%)
Osteopenia	9	1	10	90	10
Normal bone mass	0	10	10	100	0

The specificity of the model is 100 %, the sensitivity was 90 % by leave-one-out cross validation test

### Validation of the candidate biomarker secretin using ELISA

The peak with m/z 3038 in the SVM model was suggested as Secretin by TagIdent tool. Enzyme-linked immunosorbent assays (ELISA) is commonly used in clinical settings, for the direct validation and quantification of the identified Secretin, the ELISA technique was used.

The 80 serums for ELISA validation from 40 osteopenia patients and 40 postmenopausal women with normal bone mass, specifically (Table 4). Statistical analysis showed that there is no bias for age, weight, height, and duration of menopause between the osteopenia group and healthy control.

**Table 4:** Comparisons of Clinical features of patients in the ELISA validation

Groups	Age (years)	Weight (kg)	Height (cm)	Duration of menopause (years)
Osteopenia (n = 40)	56.56 ± 3.78	54.09 ± 4.98	161.43 ± 10.29	5.34 ± 2.56
Normal bone mass (n = 40)	56.10 ± 3.50	53.29 ± 3.85	162.52 ± 10.58	4.95 ± 1.63
t	0.56	0.80	0.47	0.81
P values	>0.05	>0.05	>0.05	>0.05

Data shown are mean ± standard deviation

The secretin concentrations of postmenopausal women with normal bone mass and osteopenia patients differed significantly (P < 0.05). Result showed that the normal bone mass group had significantly higher secretin signals than that of osteopenia group. The result is shown in Table 5. As expected, the results of ELISA validation is consistent with the MALDI-TOF MS results.

**Table 5:** Comparisons of secretin concentrations in the ELISA validation

Groups	Secretin concentration (pg/mL)	t values	P values
Normal bone mass (n = 40)	307.48 ± 74.68	t = 8.523	<0.05
Osteopenia (n = 40)	157.41 ± 82.62		

Data shown are mean ± standard deviation

## Discussion

Although some progress has been made in the analysis of osteopenia disease, and to date there was no previous study to describe the application of MB-WCX combined with MALDI-TOF-MS technology in serum samples in osteopenia. In the present study, our results demonstrated that a diagnostic model was constructed successfully, which had discriminated postmenopausal women with osteopenia and postmenopausal women with normal bone mass.

In recent years with the development of proteomics technology, a variety of biological markers in clinical been widely applied (Siew et al. 2011; Hampel et al. 2014; Rousseau and Garnero 2012). Biological markers play an important role in disease diagnosis. Using serum proteomics technology (Ray et al. 2011) (Nanjappa et al. 2013) to find important molecular markers provides an effective method for the clinical diagnosis of the disease.

Currently, the commonly used serum bone markers, such as total Procollagen I N Terminal Peptide (PINP) (Pollmann et al. 2007), the carboxy-terminal cross-linking telopeptide of type I collagen (β-CrossLaps) (Chubb et al. 2015), N-terminal osteocalcin and 25-hydroxyvitamin D3 reflect the changes of bone metabolism. However, some bone turnover markers have a low sensitivity and specificity in the early diagnosis of osteopenia that did not meet requirements of non-invasive and specific early diagnosis of osteopenia in the clinical practice (Bhattacharyya et al. 2008).

In this study, we screened 2 discriminating peaks of 1699 and 3038 Da which had distinguished serum samples between postmenopausal women with osteopenia and postmenopausal women with normal bone mass effectively. The serum proteomic model, based on a group of 2 peaks, accurately recognized postmenopausal women with osteopenia and postmenopausal women with normal bone mass with a sensitivity of 90 % and a specificity of 100 % by Leave-one-out test verification. The peak with m/z 3038 in the SVM model was suggested as Secretin by TagIdent tool. Results also showed that the normal bone mass group had significantly higher secretin signals than that of osteopenia group by ELISA. Secretin was known to be produced in response to low pH in the duodenum or passage of food into the duodenum in an effort to buffer the gastric acids. Secretin was also structurally closely related to calcitonin (Kopic and Geibel 2013). Perhaps the lower secretin in the osteopenia implied less buffering of gastric acid, which could thereby inhibit calcium absorption.

For screening biomarkers, a variety of proteomic approaches have recently been performed to determine the protein composition in clinic. Most of these proteomic technologies used mass spectrometric (MS) techniques combined with several analytical techniques such as two-dimensional gel electrophoresis (2D-GE) or liquid chromatography (LC). While these approaches provided a large amount of data, they are generally very time-consuming and hence restrictive in the number of comparative samples that can be analyzed.

MB-WCX with MALDI-TOF-MS technology is an approach that offers a unique platform for high-throughput protein profiling of complex biological samples such as serum samples (Pusch and Kostrzewa 2005) (Cho et al. 2013).

Furthermore, the cost of using this technology is low, and further protein identification can be performed from the eluted material without complex purification (Wieser et al. 2012). Thus, magnetic beads-based enrichment approaches have the potential to capture and enrich low abundance, low molecular weight species (Whiteaker et al. 2007).

In addition, the advantage of MALDI-TOF-MS is the direct use of crude sample, large-scale, high-throughput, automated, and minimal sample requirements. MALDI-TOF-MS not only can find a protein or biological markers, it also detected the combination of proteins existing in different forms (Gould et al. 2004).

In this study, all aspects of data processing such as sample inclusion and exclusion, serum collection, processing and storage of serum, serum protein separation and purification, sample point target, MS acquisition, experimental methods and parameter settings were optimized. Discovery researches in the clinical samples is better than in vitro and in vivo experiments.

In the aspect of data processing, data analysis software system was used to analysis data. The noise analysis was removed using undecimated discrete wavelet transform (UDWT) analysis (Aiazzi et al. 2002; Fowler 2005). The model was established by SVM method. SVM were used to align and integrate hundreds of mass data points from large numbers of samples, and could be used to process many samples in parallel. This approach is sensitive and fast, these were features essential for clinical use. SVM exhibited many unique advantages in solving small sample, nonlinear and high dimensional pattern recognition problems and achieve the statistical theory of structural risk minimization principle. The application of these techniques obtained serum protein diagnostic model with high credibility.

## Conclusions

This study provides a new serological method for discovering serum protein markers to screen and diagnose osteopenia. This will be helpful in preventing and treating primary type I osteoporosis. Therefore, WCX magnetic beads and MALDI-TOF-MS can be used as a fast and cost-effective approach for serum samples discovery of predictive biomarkers of disease in osteopenia.

We speculate that the Secretin (SECR_HUMAN, P09683, Chain: 28–54, pI: 9.46, Mw: 3040) would be potential biomarker for forecasting osteopenia in postmenopausal women.

However, there is still a gap between our findings and their application in clinical practice. Future controlled studies with larger sample sizes are required to determine the sensitivity and specificity of secretin in the diagnosis of osteoporosis. The further validation, function, interaction, and metabolic pathways of these proteins will be the future research. Moreover, Regarding to the MALDI-TOF-MS technology, for this technology does not identify protein or peptides sequence, the identities of peaks by TagIdent may not be the indeed protein or peptides. And therefore, the purification and identify of indeed protein or peptides were needed based on the standard sequencing approaches in the further.

## Methods

### Subjects and samples collection

Serum samples were collected from consenting patients (n = 20, 10 from postmenopausal women with osteopenia plus 10 from postmenopausal women with normal bone mass) enrolled from department of orthopedics, the Second Affiliated Hospital of Zhejiang Chinese Medicine University, from June 2013 and January 2014.

### Diagnostic criteria

The diagnosis of osteopenia was based on the following recommended criteria by the WHO: Survey the lumbar vertebra normal position bone density by using dual energy X-ray absorptiometry (Unnanuntana et al. 2010; Frost et al. 2012), T score −1 to −2.5 could be diagnosed as osteoporosis [T = the standard deviation of (measured value-peak bone mass)/(normal adult bone density)].

### Inclusion criteria

Patients were included in the study based on the following criteria: (1) they conform to the diagnostic criteria of osteoporosis; (2) they were postmenopausal women; (3) they were from 50 to 60 years old.

### Exclusion criteria

Patients were excluded from the study based on the following criteria: (1) those that also had diseases that severely affect the metabolism of bone or calcium, such as diabetes, Cushing’s syndrome, function changing of the thyroid or parathyroid, osteomalacia, rheumatoid arthritis, multiple myeloma, bone tumor, osteoarthrosis, Paget’s disease, and osteogenesis imperfecta; (2) those that also had severe primary cardiac diseases, or diseases of the cerebral vessels or hematopoietic system; (3) those that also had severe liver function or renal insufficiencies; (4) those taking drugs within the past 6 months that affect bone metabolism, such as estrogen, steroid hormones, calcitonin, parathyroid hormones, bisphosphonates, fluoride, vitamin D, anticonvulsant drugs, and diuretics; (5) those who had a medical history of mental illness; and (6) patients with Alzheimer’s disease.

### Ethical review

This study was approved by the local Ethics Committee of The Second Affiliated Hospital of Zhejiang Chinese Medicine University. The patients and volunteers provided written informed consent for their participation before they enrolled in this study.

### Sample collection and preparation

The blood samples (5 ml) were collected in the morning and allowed to clot at room temperature for up to 1–2 h. The samples were then centrifuged at 4 °C for ten min at 3000 rpm (in 943×*g*). The serum were frozen and stored at −80 °C for future analysis. The length of cryopreservation period for each serum sample was less than 6 months.

### Weak cation magnetic separation technology analysis

Magnetic beads-based weak cation exchange chromatography (MB-WCX) (Bruker Daltonics, Germany) was used for peptidome separation of samples. We prepared a 200-μL sample tube of thoroughly mixed weak cation magnetic suspension by adding 10 μL magnetic beads binding buffer, 10 μL magnetic beads suspension, and 5 μL serum, mixing at least five times using the sampling gun, and standing at room temperature for 5 min. We put the sample tube into the magnetic separator, maintained the magnetic field for 1 min, used the sampling gun to absorb the liquid after separation of the magnetic beads and liquid, and added to the sample tube 100 μL magnetic beads cleaning buffer. We then moved the sample tube ten times between the two adjacent holes of the magnetic separator, left to stand, and used the sample gun to absorb the supernatant again after magnetic beads adherence. We repeated the cleaning process twice. We then took down the sample tube from the magnetic separator, added 5 μL magnetic beads elution buffer to the sample tube, and repeated the pipetting. We put the sample tube into the magnetic separator and let it stand for 2 min, transferred the supernatant to a clean 0.5-mL sample tube when the magnetic beads were completely adhered, then added 5 μL magnetic beads stabilizing buffer. The eluate was then ready for spotting onto MALDI-TOF MS targets and measured.

### Anchor chip spotting

1 μL eluted sample was spotted onto a MALDI-TOF AnchorChipTM target (600 μm anchor diameter) and air-drying at room temperature, then 1 μL matrix (0.3 mg/mL HCCA, 50 %ACN, 2 %TFA) was spotted onto MALDI-TOF AnchorChipTM target.

### MALDI-TOF MS

AnchorChipTM target was placed into the Microflex mass spectrometer (Bruker Daltonics). Samples were detected after calibration of the instrument by ClinProt standard (Bruker Daltonics). MALDI-TOF MS parameters are detailed in Additional file 1.

### Detection data analysis

The Zhejiang University-ProteinChip data analysis system (ZJU-PDAS) software, designed at Zhejiang University Cancer Institute, was used to analyze the raw data (Shi et al. 2014). The process was as follows. (1) The original spectrum was uploaded to the server to map data for protein analysis, and to process homogeneous data. (2) We removed the mass-to-charge ratio (m/z) of raw data below peak 2000, used the UDWT analysis method to remove the noise caused by the mass spectrometer itself, and used the amended data after the removal of the baseline noise spectrum and correction of the molecular weight values map. (3) We identified the protein charge ratio peaks with local minima, and filtration peak signal-to-noise ratios of less than 3. The differences in m/z between each sample of <0.3 % of the peak were clustered together. After clustering, peaks appeared only in the sample in less than 10 % removed. (4) To find the peak intensity in the respective sample treated as uniform. (5) After pretreatment, we filtered out protein charge ratio peaks using the Wilcoxon rank sum test, and screened differential protein peaks (P < 0.05). (6) We screened a random combination of different proteins, and used a genetic arithmetic model and the support vector machine (SVM) computing model to establish serum protein fingerprints.

For the SVM, we used the radial basis function, a gamma value of 0.6, and the penalty function (C) was set to 19. The highest elected combinations were used to build the SVM model to predict the Youden index. Because the final candidate signs matter, we used the leave-one-assessment model to predict the effect of an established law and the final results were cross-validated.

ZJU-PDAS specific parameters of analysis were detailed in Additional file 2.

### Identities of the two differential peaks suggested by TagIdent

Using TagIdent tool (Washam et al. 2013; Liu et al. 2012) network software (http://web.expasy.org/tagident/), molecular weight (Mw) range was set to 0.3 %, Isoelectric point (PI) range value was set to min = 4, max = 14. Checked for protein sequences with cysteines oxidized (–SS–); Organism name: *homo sapiens*; Tagging: Display only the sequences matching the tag, Displayed the predicted N-terminal sequence, Databases on UniProtKB/Swiss-Pro. Proteins as candidate were selected by the most similar molecular weight.

### Immunoassay for quantification of the candidate biomarker secretin

After the identity was suggested by TagIdent, ELISA was used to detect the levels in sera. Secretin levels were measured in the serum samples using an ELISA 96-well plate for secretin (CEB075Hu, Cloud-Clone Corp., Houston, USA). The serum samples were diluted 1:1 with a 0.01 mol/L PBS (PH 7.0–7.2) according to the manufacturer’s instructions and analyzed on a RT-2100C micro plate reader (Rayto, USA) at a wavelength of 450 nm according to the manufacturer’s instructions.

### Statistical analysis

All MALDI-TOF-MS spectrum were analyzed by ZJU-PDAS software to detect the peak intensities of interest, and to compile the peaks across the spectrum obtained from serum samples. All statistical comparisons were done by SPSS software version 13.0 (SPSS Inc., USA). The comparison of the age, weight, height, duration of menopause, and comparisons of secretin concentrations was done by t test, test level of α = 0.05. The Wilcoxon rank sum test (test level of α = 0.05) was used to compare features. P values <0.05 were considered statistically significant. Genetic algorithm (GA) (Deb et al. 2002), Support vector machine (SVM) (Weston et al. 2000), and Youden Index (Schisterman et al. 2005) methods were used to establish the diagnosis model and predict the candidate markers. The model was verified by leaving-one-out cross validation test (Wuebker et al. 2010).

## Additional files

10.1186/s40064-016-2276-4 Detection parameters of Microflex MALDI-TOF-MS instrument.
10.1186/s40064-016-2276-4 Specific parameters of Zhejiang University-ProteinChip data analysis system (ZJU-PDAS).

## Abbreviations

GA: genetic algorithm
MB-WCX: weak cation exchange magnetic beads
MALDI-TOF-MS: matrix-assisted laser desorption ionization-time of flight-mass spectrometry
MS/MS: tandem mass spectrometry
DXA: dual-energy X-ray
BMD: bone mineral density
UDWT: undecimated discrete wavelet transform
SVM: support vector machine

## References

[CR1] Aiazzi B, Alparone L, Baronti S, Garzelli A (2002). Context-driven fusion of high spatial and spectral resolution images based on oversampled multiresolution analysis. IEEE Trans Geosci Remote Sens.

[CR2] Bantis LE, Nakas CT, Reiser B (2014). Construction of confidence regions in the ROC space after the estimation of the optimal Youden index-based cut-off point. Biometrics.

[CR3] Bhattacharyya S, Siegel ER, Achenbach SJ, Khosla S, Suva LJ (2008). Serum biomarker profile associated with high bone turnover and BMD in postmenopausal women. J Bone Miner Res.

[CR4] Cho Y-T, Su H, Huang T-L, Chen H-C, Wu W-J, Wu P-C, Wu D-C, Shiea J (2013). Matrix-assisted laser desorption ionization/time-of-flight mass spectrometry for clinical diagnosis. Clin Chim Acta.

[CR5] Chubb SP, Mandelt CD, Vasikaran SD (2015). Comparison of results from commercial assays for plasma CTX: the need for harmonization. Clin Biochem.

[CR6] Consensus NIH (2001). Development panel on osteoporosis prevention, diagnosis, and therapy. Osteoporosis prevention, diagnosis, and therapy. JAMA.

[CR7] Czerwiński E, Badurski J, Marcinowska-Suchowierska E, Osieleniec J (2006). Current understanding of osteoporosis according to the position of the World Health Organization (WHO) and International Osteoporosis Foundation. Ortop Traumatol Rehabil.

[CR8] Deb K, Pratap A, Agarwal S, Meyarivan T (2002). A fast and elitist multiobjective genetic algorithm: NSGA-II. IEEE Trans Evol Comput.

[CR9] Fan NJ, Gao CF, Wang XL, Zhao G, Liu QY, Zhang YY, Cheng BG (2012). Serum peptidome patterns of colorectal cancer based on magnetic bead separation and MALDI-TOF mass spectrometry analysis. J Biomed Biotechnol.

[CR10] Fluss R, Faraggi D, Reiser B (2005). Estimation of the Youden Index and its associated cutoff point. Biom J.

[CR11] Fowler JE (2005). The redundant discrete wavelet transform and additive noise. IEEE Signal Process Lett.

[CR12] Frost M, Gudex C, Rubin K, Brixen K, Abrahamsen B (2012). Pattern of use of DXA scans in men: a cross-sectional, population-based study. Osteoporos Int.

[CR13] Gould KL, Ren L, Feoktistova AS, Jennings JL, Link AJ (2004). Tandem affinity purification and identification of protein complex components. Methods.

[CR14] Hampel H, Lista S, Teipel SJ, Garaci F, Nistico R, Blennow K, Zetterberg H, Bertram L, Duyckaerts C, Bakardjian H (2014). Perspective on future role of biological markers in clinical therapy trials of Alzheimer’s disease: a long-range point of view beyond 2020. Biochem Pharmacol.

[CR15] Kanis J, McCloskey E, Johansson H, Cooper C, Rizzoli R, Reginster JY (2013). European guidance for the diagnosis and management of osteoporosis in postmenopausal women. Osteoporos Int.

[CR16] Knapp K, Blake G, Spector T, Fogelman I (2004). Can the WHO definition of osteoporosis be applied to multi-site axial transmission quantitative ultrasound?. Osteoporos Int.

[CR17] Kopic S, Geibel JP (2013). Gastric acid, calcium absorption, and their impact on bone health. Physiol Rev.

[CR18] Liu C, Pan C, Shen J, Wang H, Yong L (2012). Identification of serum amyloid A in the serum of gastric cancer patients by protein expression profiling. Oncol Lett.

[CR19] Nanjappa V, Thomas JK, Marimuthu A, Muthusamy B, Radhakrishnan A, Sharma R, Khan AA, Balakrishnan L, Sahasrabuddhe NA, Kumar S (2013). Plasma Proteome Database as a resource for proteomics research: 2014 update. Nucleic Acids Res.

[CR20] Oei L, Rivadeneira F, Ly F, Breda SJ, Zillikens MC, Hofman A, Uitterlinden AG, Krestin GP, Oei EH (2013). Review of radiological scoring methods of osteoporotic vertebral fractures for clinical and research settings. Eur Radiol.

[CR21] Pollmann D, Siepmann S, Geppert R, Wernecke K-D, Possinger K, Lüftner D (2007). The amino-terminal propeptide (PINP) of type I collagen is a clinically valid indicator of bone turnover and extent of metastatic spread in osseous metastatic breast cancer. Anticancer Res.

[CR22] Pusch W, Kostrzewa M (2005). Application of MALDI-TOF mass spectrometry in screening and diagnostic research. Curr Pharm Des.

[CR23] Qiu F-M, Yu J-K, Chen Y-D, Jin Q-F, Sui M-H, Huang J (2009). Mining novel biomarkers for prognosis of gastric cancer with serum proteomics. J Exp Clin Cancer Res.

[CR24] Ray S, Reddy PJ, Jain R, Gollapalli K, Moiyadi A, Srivastava S (2011). Proteomic technologies for the identification of disease biomarkers in serum: advances and challenges ahead. Proteomics.

[CR25] Rousseau JC, Garnero P (2012). Biological markers in osteoarthritis. Bone.

[CR26] Schisterman EF, Perkins NJ, Liu A, Bondell H (2005). Optimal cut-point and its corresponding Youden Index to discriminate individuals using pooled blood samples. Epidemiology.

[CR27] Shi X, Li C, Liang B, He K, Li X (2014). Weak cation magnetic separation technology and MALDI-TOF-MS in screening serum protein markers in primary type I osteoporosis. Genet Mol Res.

[CR28] Siew ED, Ware LB, Ikizler TA (2011). Biological markers of acute kidney injury. J Am Soc Nephrol.

[CR29] Unnanuntana A, Gladnick BP, Donnelly E, Lane JM (2010). The assessment of fracture risk. J Bone Joint Surg.

[CR30] Washam CL, Byrum SD, Leitzel K, Ali SM, Tackett AJ, Gaddy D, Sundermann SE, Lipton A, Suva LJ (2013). Identification of PTHrP (12–48) as a plasma biomarker associated with breast cancer bone metastasis. Cancer Epidemiol Biomark Prev.

[CR31] Weston J, Mukherjee S, Chapelle O, Pontil M, Poggio T, Vapnik V (2000) Feature selection for SVMs. In: NIPS. Citeseer, pp 668–674

[CR32] Whiteaker JR, Zhao L, Zhang HY, Feng L-C, Piening BD, Anderson L, Paulovich AG (2007). Antibody-based enrichment of peptides on magnetic beads for mass-spectrometry-based quantification of serum biomarkers. Anal Biochem.

[CR33] Wieser A, Schneider L, Jung J, Schubert S (2012). MALDI-TOF MS in microbiological diagnostics—identification of microorganisms and beyond (mini review). Appl Microbiol Biotechnol.

[CR34] Wu S, Xu K, Chen G, Zhang J, Liu Z, Xie X (2012). Identification of serum biomarkers for ovarian cancer using MALDI–TOF-MS combined with magnetic beads. Int J Clin Oncol.

[CR35] Wuebker J, Mauser A, Ney H (2010) Training phrase translation models with leaving-one-out. In: Proceedings of the 48th annual meeting of the association for computational linguistics. Association for Computational Linguistics, pp 475–484

[CR36] Zhang X, Yuan Z, Shen B, Zhu M, Liu C, Xu W (2012). Discovery of serum protein biomarkers in rheumatoid arthritis using MALDI-TOF-MS combined with magnetic beads. Clin Exp Med.

[CR37] Zheng N, Pan C, Liu W (2011). New serum biomarkers for detection of endometriosis using matrix-assisted laser desorption/ionization time-of-flight mass spectrometry. J Int Med Res.

