# Supplementary material for: Weak cation exchange magnetic beads coupled with matrix-assisted laser desorption ionization-time of flight-mass spectrometry in screening serum protein markers in osteopenia
Source: Springerplus. 2016 May 21;5(1):679. doi: 10.1186/s40064-016-2276-4 (PMC4899343; doi:10.1186/s40064-016-2276-4)
Supplement: Supplementary file 1 — 10.1186/s40064-016-2276-4 Detection parameters of Microflex MALDI-TOF-MS instrument. [file 40064_2016_2276_MOESM1_ESM.doc]

**Supplementary materials 1**. *Matrix-assisted laser desorption ionization-time of flight-mass spectrometry (MALDI-TOF-MS)*

*Detection parameters of Microflex MALDI-TOF-MS instrument*

| **Parameters** |  |
| --- | --- |
| **N_2_ pressure** | approx. 1700–2000 mbar |
| **Laser** | First, bombardment with 10–20 shots of high laser energy, then, in the same position, bombardment with 40–50 shots with energy acquisition lower laser energy of 10–20%. |
| **Parameters** | **Autoflex 1–10 kDa** |
| **Shots** | 8–10 different crystallizations at the same target point, in the 400–500 shots |
| **Spectrometer** |  |
| Ion source 1 | 20 kV |
| Ion source 2 | 18.4 kV |
| Lens | 7.5 kV |
| Pulsed ion extraction | 120 ns |
| Polarity | positive |
| Matrix suppression mode | gating |
| Gating strength | high/maximum |
| Suppress up to | approx. 800 Da |
| **Detection** |  |
| Mass range | low: 900–10,500 Da |
| Detector gain | 1600–1800 V |
| Sample rate | 1.00 |
| Electronic gain | Regular, 100 mV |
| Real time smooth | high |
| Laser frequency | 25 Hz |
| Laser attenuator | e.g., 60/30 |
