# Supplementary material for: Weak cation exchange magnetic beads coupled with matrix-assisted laser desorption ionization-time of flight-mass spectrometry in screening serum protein markers in osteopenia
Source: Springerplus. 2016 May 21;5(1):679. doi: 10.1186/s40064-016-2276-4 (PMC4899343; doi:10.1186/s40064-016-2276-4)
Supplement: Supplementary file 2 — 10.1186/s40064-016-2276-4 Specific parameters of Zhejiang University-ProteinChip data analysis system (ZJU-PDAS). [file 40064_2016_2276_MOESM2_ESM.doc]

**Supplementary materials 2.** Zhejiang University-ProteinChip data analysis system (ZJU-PDAS) analysis of specific parameters.

| **User name** |  | **Peaks filtering factor** | **Wilcoxon** |
| --- | --- | --- | --- |
| Repeat sample | 0 | Small size sample | No |
| Test sample percent | 0.3 | Batch analyze | 1 |
| Wavelet threshold | 50 | Smoothing window | 100 |
| Calibration coefficient (%) | 0.03 | Minimal m/z (%) | 2000 |
| Cluster factor (%) | 0.003 | Minimal peak threshold (%) | 0.1 |
| Excluded spectra threshold (%) | 0.2 | Minimal signal/noise ratio (%) | 3 |
| Minimal intensity | 300 | P-value or number of peaks | 22 |
| Algorithm | 1 | Evaluation | 2 |
| GA population | 50 | GA generation | 20 |

GA: genetic arithmetic; m/z: mass-to-charge ratio
